# Green synthesis of magnesium oxide nanoparticles using *Nigella sativa* seed extract: characterization, *in vitro* antioxidant activities, and wound healing efficacy in streptozotocin-induced diabetic rats

**DOI:** 10.1039/d5ra07329d

**Published:** 2026-02-10

**Authors:** Awatef Elwej, Abir Mhadhbi, Manel Mohammed alzahrani, Hassane oudadesse, Hamadi Fetoui, Naceur Mejri, Samira Jebahi

**Affiliations:** a Laboratory of Toxicology and Environmental Health. LR17ES06, Sciences Faculty of Sfax, University of Sfax Sfax BP1171 3000 Tunisia; b Research Laboratory on Energy and Matter for Nuclear Science Development (LR16CNSTN02), National Center for Nuclear Sciences and Technologies Tunis Tunisia jbahisamira@yahoo.fr; c Chemistry Department, Faculty of Science, Al-Baha University Saudi Arabia; d University of Rennes, CNRS, ISCR-UMR 6226 F-35000 Rennes France; e Laboratory of Biotechnology and Nuclear Technology, National Center for Nuclear Sciences and Technologies Tunis Tunisia

## Abstract

This study investigated the green synthesis of magnesium oxide nanoparticles (MgO-NPs) using *Nigella sativa* (*N. Sativa*) seed extract and evaluated their antioxidant and wound healing properties in diabetic rats. *N. sativa* seeds (black cumin) were extracted with distilled water, and this extract was then used for the biosynthesis of MgO-NPs, characterized by a color change and subsequent centrifugation and drying. The successful formation and characteristics of the nanoparticles were confirmed using UV-Vis spectrophotometry, UHPLC-ToF-MS analysis, Fourier-transform infrared (FTIR) spectroscopy and scanning electron microscopy (SEM). The synthesized MgO-NPs were assessed for their *in vitro* antioxidant activity using reducing power and ABTS radical scavenging assays. Furthermore, the *in vivo* wound healing efficacy was examined in streptozotocin-induced diabetic Wistar rats. An excision wound model was established, and MgO-NPs were topically applied daily. Wound contraction was monitored over 17 days, and biochemical parameters such as C-reactive protein, lipid peroxidation, advanced oxidation protein products, GSH, non-protein thiols, and antioxidant enzyme activities (CAT, SOD, GPx) were measured in serum and tissue samples. As a result, a significant acceleration of wound healing was observed in diabetic rats treated with MgO-NPs, as evidenced by a reduction in wound diameter and decreased CRP levels (−22%) compared to the control group, as well as a modulation of enzymatic and non-enzymatic antioxidants, which was further confirmed by histological analysis. Considering our outcomes, MgO-NPs could be used as an effective dressing material as well as a tissue regrowth scaffold for diabetic wound therapy.

## Abstract 2

This study investigated the green synthesis of magnesium oxide nanoparticles (MgO-NPs) using *Nigella sativa* (*N. Sativa*) seed extract and evaluated their antioxidant and wound healing properties in diabetic rats.

## Graphical abstract

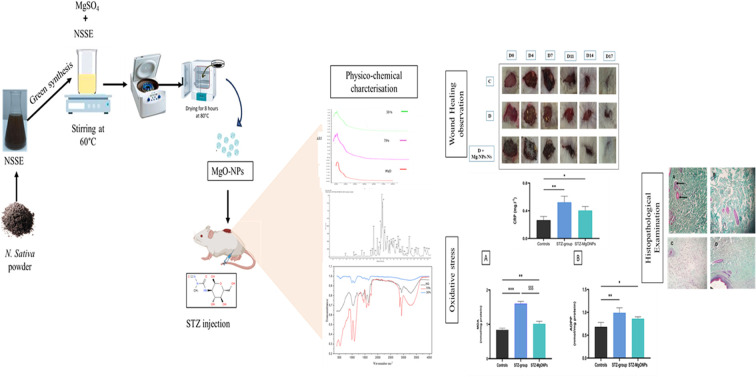

## 1. Introduction

Nanotechnology has become one of the most promising applications^1^ for developing novel nanomedicine-based strategies in the effective management of diabetes and its associated complications.^2^ Nanoparticles (NPs) are central to numerous technological advancements due to their unique physicochemical properties, such as high surface area-to-volume ratios and enhanced reactivity, which differ markedly from their bulk counterparts.^3^ These characteristics enable transformative applications notably in the biomedical field, particularly as versatile tools in targeted drug delivery,^4^ regenerative medicine^5^ and in the treatment of various diseases, such as cancer.^6^ However, the synthesis of these nanomaterials often relies on conventional physical and chemical methods that necessitate high temperatures, vacuum conditions, sophisticated instruments, and chemical additives, and raise serious concerns due to the use of toxic chemicals that increase risks to environmental safety and human health.^7^ Thus, the great interest of the scientific community is particularly focused on the synthesis of nanomaterials using biogenic sources such as microorganisms,^8^ algae^9^ and different parts of plants.^10^ Due to their safety and eco-friendly nature, recent green synthesis methods of nanomaterials have emerged as a promising approach combining rapid production, cost efficiency, and environmental sustainability.^11^ Wound management has seen considerable benefits where nanoparticles are increasingly invested to enhance healing processes by improving drug penetration. Despite progress, the effective treatment of chronic wounds, especially those associated with systemic conditions like diabetes mellitus, remains a clinical challenge. Diabetic wounds suffer from a complex, multifactorial impairment of the normal healing cascade, often involving chronic inflammation, heightened oxidative stress and poor angiogenesis, requiring more advanced therapeutic approaches.^12^ These approaches are favored due to the biocompatibility and the interaction of nanoparticles with bioactive phytochemical compounds of plants, potentially enhancing their therapeutic effects.^13^ Concurrently, magnesium (Mg), an essential trace element, plays vital roles in cellular metabolism, inflammation regulation, and tissue regeneration, making it an attractive agent for the synthesis of magnesium-based nanoparticles (MgO-NPs). *Nigella sativa* (black seed) is well-documented for its potent antioxidant, anti-inflammatory,^14^ and wound healing properties,^15^ making its extract an ideal candidate for green synthesis protocols.

To the best of our knowledge, this is the first study to investigate diabetic wound healing through the application of MgO-NPs green-synthesized using *Nigella sativa* seed extract. Therefore, this study aimed to synthesize and characterize *Nigella sativa*-derived MgO-NPs and subsequently evaluate their therapeutic efficacy in promoting wound closure and tissue regeneration in an established *in vivo* diabetic rat model.

## 2. Materials and methods

### 2.1. Plant material

*N. sativa* (black cumin) seeds were obtained from the local market. The various chemicals used in the research work were of analytical grades such as magnesium sulphate (MgSO_4_·7H_2_O), sodium hydroxide (NaOH) and hydrochloric acid (HCl) were procured from Sigma Aldrich, St. Louis, MO, USA of A.R. grade.

### 2.2. Preparation of *N. sativa* seed extract

The seeds of *N. sativa* were first washed several times then they were dried at room temperature. After the seeds were allowed to dry for a specified duration, they were ground into a fine powder (50 g) and dissolved in distilled water (250 mL). The mixture was continuously stirred and heated (60 °C), for 30 minutes under constant agitation. Finally, the extract of *N. sativa* seeds (NSSE) was collected and filtered in Whatman no. 1 to be stored (4 °C) in dark for further use.

### 2.3. Green synthesis of MgO-NPs

The biosynthesis of MgO-NPs using *N. sativa* seed extract was outlined according to.^16^ In brief, 50 mL of 10 mM magnesium sulfate solution was added drop by drop to 10 mL of NSSE under stirring at 300 rpm for 60 min. The pH of the solution was adjusted to 9 by adding NaOH. The color change from yellowish green to brown was obtained. After storing it at room temperature in dark flask, the solution was centrifuged (5000 rpm for 20 min) and washed with deionized water three times and dried at 70 °C in a hot air oven overnight. After drying, MgO-NPs were scratched and stored in Eppendorf tube for further use.

### 2.4. UV-visible spectroscopy

A UV-Visible spectrophotometer (UV-1601 PC, Shimadzu, Japan; H14 grating (UV through shortwave NIR with optical resolution of 0.4 nm)) was used to perform UV-visible characterisation. Quartz cuvettes with a 1 cm path length were used to measure absorbance over the 250–700 nm wavelength range. Prior to each usage, the cuvettes were cleaned by sonicating them for 5 minutes in deionized water and then rinsing them with the same water.

### 2.5. UHPLC-ToF-MS conditions

Concerning the chromatographic conditions, water [A] and acetonitrile [B], both acidified with 0.1% formic acid, constituted the mobile phase. The gradient program used, with a total time of 15 min, was as follows: 0–1 at 25% [B]; 1–13 min from 25% to 95% [B] and maintained for 2 min; and back to 10% [B] in 1 min, with a flow rate of 0.3 mL min^−1^. The separation of compounds was carried out through an Acquity UPLC equipped with HSS T3 C18 (2.1 mm × 100 mm, 1.8 µm) column at (30 °C). The volume of injection was 20 µL, while the autosampler was held at 4 °C. The acquisition was carried out in full scan from 100 to 750 Da using the following mass spectrometry parameters: ion source voltage: 5500 V; source temperature: 120 °C; curtain gas (CUR): 30 psi; gas 1 and gas 2: 55 psi; and declustering potential (DP): 100 V. The software used for this analysis was Analyst® TF (SCIEX, Foster City, CA, USA, version 1.7). For the identification, PeakView™, LibraryView™ was used.

### 2.6. Fourier transform infrared spectroscopy analysis (FTIR)

Fourier transform infrared spectroscopy was carried out using a Bruker spectrometer model Tensor. The measurement range was 4000 to 400 cm^−1^, with a resolution of 4 cm^−1^ and 32 scans for the sample and background. The samples were prepared by combining 0.0020 g of the powders with 0.20 g of KBr and compressed with a PIKE Technologies Crush IR hydraulic press equipment at 9.9 tons of pressure for 1 minute. The compacted sample was then analyzed using FTIR instruments.

### 2.7. Scanning electron microscope analysis (SEM)

Utilizing a Zeiss Sigma 300 scanning electron microscope (SEM) (Zeiss Co., Oberkochen, Germany), the powder's surface morphology and microstructure were investigated. Samples were frozen in liquid nitrogen and then covered with gold sputtering before being viewed under a scanning electron microscope. A 15 kV accelerating potential was used.

### 2.8. *In vitro* antioxidant assays

#### 2.8.1. Ferric reducing power (FRAP) assay

The reducing power of MgO-NPs was determined using the method of Pulido *et al.*^17^ A 500 µl aliquot of MgO-NPs or of butylated hydroxy anisole (BHA) (0.06–1 mg mL^−1^), used as a standard reference, was mixed with 1 mL of phosphate buffer (0.2 M, pH 6.6) and 1 mL of 1% potassium ferricyanide [K_3_Fe(CN)_6_]. The solution was then incubated at 50 °C for 20 min. After that, 1 mL of 10% trichloroacetic acid (TCA) was added to the mixture and centrifuged at 3000 rpm for 15 min. The resulted supernatant was combined with distilled water and ferric chloride (FeCl_3_, 0.1%). The absorbance was measured spectrophotometrically at 700 nm. Results were expressed as BHA equivalents (BHA.E) per mg of MgO-NPs (mg GAE/mg MgO-NPs).

#### 2.8.2. ABTS assay

The radical scavenging activity of synthesized MgO-NPs was determined.^18^ 2.2-azino-bis(3-ethylbenzothiazoline-6-sulfonic acid) (ABTS) (7 mM) and potassium persulfate (2.45 mM) solutions were mixed and stored in a dark room for 12–16 h before to use. Before the analysis, the ABTS solution was diluted with ethanol to an absorbance of 0.700 ± 0.05 at 734 nm. Following the addition of 4.5 mL of the ABTS reaction mixture to the various concentrations (50–250 µg mL^−1^) of MgO-NPs (1 mg mL^−1^), the reaction mixture was vortexed. After keeping at room temperature for 15 min, the absorbance of the samples was read at 734 nm. The results were assessed as IC50 values.

### 2.9. Animals and experimental design

#### 2.9.1. Animals

Central Pharmacy (SIPHAT, Tunisia) provided Female Wistar rats (180–200 g) which were acclimatized in a room with relative humidity and temperature of 40% and 22 ± 2 °C, under a 12 hour light–dark phase. All of the rats were treated in conformity with the General Guidelines on the Use of living Animals in Scientific Investigations (1986) and approved by the Committee for the Care and Use of Laboratory Animals (Approval no. 1205).

#### 2.9.2. Diabetes induction

After overnight fasting, DM was induced in the rats by a single intraperitoneal injection of freshly prepared STZ at a dose of 60 mg kg^−1^ bw, dissolved in ice-cold saline. The rats were allowed to drink 5% glucose solution *ad libitum* overnight post STZ-injection to prevent hypoglycemia and mortality. After sufficient time of STZ injection (72 days), the fasting blood glucose (FBG) levels were assessed by collecting samples from the tail vein and measuring them with an Accu-Chek Performa meter (USA). Rats with high glucose level (≥250 mg dl^−1^) along with glycosuria and hyperglycemia, were considered as diabetics and therefore used for the experiment.

#### 2.9.3. Wound model

Excision model was used for the evaluation of wound contraction in this protocol. Animals in each group were anesthetized by hydrate chloral. Briefly, hairs on the dorsum of diabetic and normal nondiabetic rats were shaved, the exposed skin area was cleaned with 70% ethanol, then the circular wound (diameter = 2 cm) was created on the dorsal region of each animal by excising the skin. The wounds were left open without any dressing material for the duration of the experiment study. Wound healing was monitored by taking photographs on 0, 4, 7, 11, 14, and 17 days after wounding.

#### 2.9.4. Experimental design

The experimental procedure was conducted as follows: after wound creation, rats were randomly allocated into three groups: group (1) non-diabetic normal group served as the controls and treated with a saline solution (0.9%); group (2) diabetic control rats treated with a saline solution (0.9%); group (3) diabetic treated rats by a one topical application of MgO-NPs in each excision wound of the rats once per day, until the sacrifice. Prior to application, the MgO-NPs suspension was sonicated to disperse aggregates and achieve homogeneous distribution. Subsequently, MgO-NPs nanoparticles were dispersed in sterile 0.9% NaCl solution to obtain a final concentration of 5 mg mL^−1^. Then the solution was applied topically to each wound site once daily using a syringe and distributed evenly with a glass rod. Rats were individually housed, maintained on normal food and water *ad libitum*, and those which showed infection signs were separated and excluded from the study and replaced.

#### 2.9.5. Wound contraction

Rats were photographed at the time of wounding (day 0) before treatment with MgO-NPs and the wound area was measured instantly by placing a transparent tracing paper over the wound and tracing it out using a permanent marker.^19^ The tracing paper was then canned to calculate the wound surface areas (WSA) with the software AUTOCAD. The wound area was estimated on different days (0th, 4th, 7th, 11th, 14th, and 17th days) and the percentage of wound contraction was calculated using: % wound contraction = (*A*_0_ − *A*_*t*_)/*A*_0_ × 100where *A*_0_ is the original wound area and at is the area of wound at specific time period after wounding.^20^

#### 2.9.6. Sampling

At the end of the 17 day study period, animals were euthanized by cervical decapitation. Whole blood samples were immediately collected into serum vacutainer tubes, then centrifuged at 2200×*g* for 10 min at 4 °C and finally stored at −80 °C until biochemical analysis.

#### 2.9.7. C-reactive protein (CRP) assessment

C-reactive protein (CRP) was analyzed by electronic automate coulter MAXM (Beckman Coulter, Inc., Fullerton, CA).

#### 2.9.8. Estimation of lipid peroxidation

Lipid peroxidation was assessed using the method outlined by Draper and Hadley,^21^ measuring absorbance at 532 nm. The results were expressed as nM of TBARS per milligram protein.

#### 2.9.9. Advanced oxidation protein products (AOPP) content

As common markers of protein oxidation, the levels of advanced oxidation protein products was measured in stomach homogenate tissues^22^ to identify the level of oxidative stress. Briefly, the stomach AOPP level was determined spectrophotometrically, and the absorbance of each sample was measured at 340 nm using the extinction coefficient of 26 000 M^−1^ cm^−1^.

#### 2.9.10. Determination of GSH levels

The GSH content was assayed according to the method of Ellman^23^ modified by Jollow *et al.*^24^ Results were expressed as µg mg^−1^ tissue.

#### 2.9.11. Determination of non-protein thiol levels

Non-protein thiol (NPSH) levels were determined by the method by Ellman^23^ and the results were expressed as µmol mg^−1^ tissue.

#### 2.9.12. Determination of antioxidant enzyme activities

(a) Catalase (CAT) activity was estimated according to the method of Aebi,^25^ based on the hydrolysis of H_2_O_2_ and the resulting decrease in the absorbance at 240 nm. CAT activity was calculated in terms of µmol H_2_O_2_ consumed/min mg protein.

(b) Superoxide dismutase (SOD) activity was determined by monitoring the photochemical reduction of nitro blue tetrazolium (NBT) as described by Beauchamp and Fridovich.^26^ One unit (U) of SOD activity corresponded to the amount of enzyme required to cause 50% inhibition of NBT reduction at 560 nm. SOD activity was expressed as U mg^−1^ protein.

(c) Glutathione peroxidase (GPx) activity was measured according to Flohé and Günzler.^27^ The enzyme activity was expressed as nmol of GSH oxidized/min mg protein.

#### 2.9.13. Histological studies

For histological examination, the excision skin tissues from all animals were removed from control and treated rats at the end of the healing phase. They were fixed in 10% buffered formalin solution and then embedded in paraffin. Sections of 5 µm thickness were placed on slides and stained with trichrome staining for histological evaluation under light microscopy.

### 2.10. Statistical analysis

The data were analyzed utilizing GraphPad Prism 10.4.0 (GraphPad Software, San Diego, CA) and conferred as mean ± SEM. Statistical differences were attended using one-way analysis of variance (ANOVA), followed by Tukey's post hoc test.

## 3. Results and discussion

### 3.1. Chemical characterization and *in vitro* analysis of MgO-NPs

#### 3.1.1. UV-visible spectroscopy analysis

**Fig. 1:**
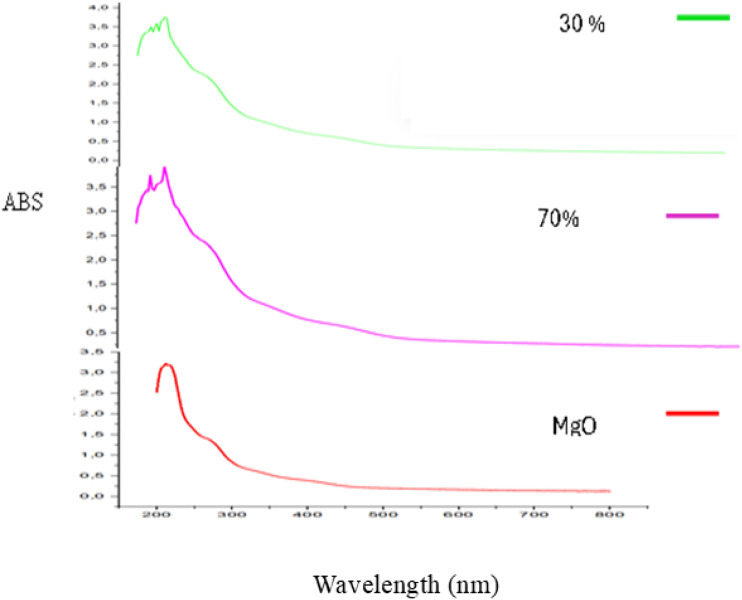
The UV-Vis spectra of biosynthesized MgO-NPs.

The UV-Vis spectra of biosynthesized MgO-NPs using the seed aqueous extract of *Nigella sativa* is shown in Fig. 1. An absorption peak at 210 nm was observed, corresponding to a characteristic absorption of MgO, as previously reported.^28^ Additionally, a distinct peak at 265 nm was detected in the UV spectrum of MgO-NPs nanoparticles.

#### 3.1.2. UHPLC-ToF-MS conditions analysis

**Fig. 2:**
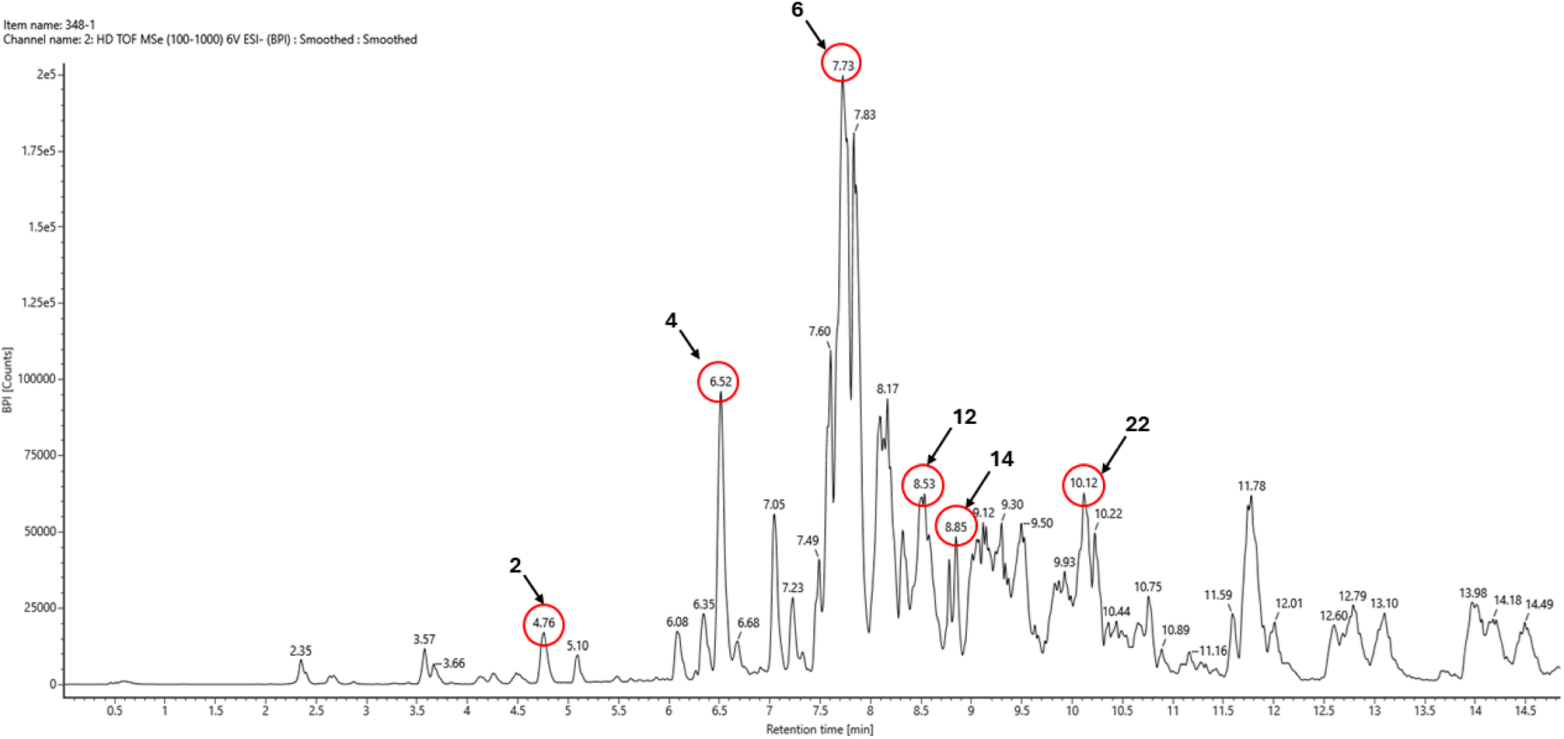
UHPLC-ToF-MS Analysis of *Nigella sativa* seed extract.

Phytochemical screening was performed by UHPLC-ToF-MS that showed the presence of biomolecules and different chemical compounds in the *Nigella sativa* seed extract. Table 1 showed the presence of flavonoid, glycolipid, and other different types of components. UHPLC-ToF-MS analysis of *Nigella sativa* seed extract previously identified several bioactive compounds, including solasonine, quercetin-*O*-α-rhamnosyl-glucopyranoside, stigmasterol 3-palmitate, 8-*O*-cinnamoylneoline, thymoquinol glucoside, magnoline, kaempferol, and trichosanic acid, vitamin D2_1 (Fig. 2).

**Table 1:** Identified compounds by UHPLC-ToF-MS in *Nigella sativa* seed extract

	Component name	Neutral mass (Da)	Neutral mass (Da)	RT (min)		Component name	Neutral mass (Da)	Neutral mass (Da)	RT (min)
1	Quercetin-*O*-α-rhamnosyl-glucopyranoside	771.20621	771.2045	2.4	23	Solanine deglycosylation	397.33447	397.3275	10.14
2	4-Hydroxymephenytoin	234.10044	234.0956	4.76	24	Vitamin D2_1	396.33922	396.3246	10.16
3	Solasonine	883.49294	883.497	6.09	25	Octadecenoic acid	282.25588	282.2579	10.26
4	Dibutyl decanedioate	314.24571	314.2511	6.52	26	Linoleyl acetate	308.27153	308.2732	10.23
5	Pingbeimine C	477.30904	477.2866	7.62	27	Octadecenoic acid	282.25588	282.2579	10.26
6	Thymoquinol glucoside	325.21949	325.2241	7.73	28	Vitexifolin C	284.21402	284.2379	10.28
7	(Z.11S)-11-Hydroxyoctadec-9-enoic acid	298.25079	298.2555	7.86	29	2-Hydroxytetracosanoic acid	384.36035	384.3608	10.36
8	14-*O*-Acetylneoline	479.2883	479.3018	8.11	30	Methyl oleate	296.27153	296.2732	10.47
9	12.13-Epoxy-9-octadecenoic acid	296.23514	296.2394	8.13	31	Eicosadienoic acid	308.27153	308.2733	10.49
10	8-*O*-Cinnamoylneoline	567.3196	567.3532	8.15	32	Stearic acid	284.27153	284.2737	10.66
11	Maristeminol	322.25079	322.2542	8.33	34	11-Eicosenoic acid	310.28718	310.289	10.76
12	Magnoline	596.28864	596.2935	8.53	35	Eicosanedioic acid	342.27701	342.2799	10.77
13	Kaempferol	285.21402	285.2017	8.65	36	Stigmasterol 3-palmitate	650.60018	650.506	11.27
14	Trichosanic acid	278.22458	278.2271	8.85	37	Eclalbasaponin VII	622.44447	622.4404	11.43
15	7.10-Octadecadienoic acid	280.24023	280.2426	9.04	38	Lophenol	400.37052	400.356	11.62
16	Octadecadienoic acid	280.24023	280.2423	9.27	39	Feruloy ampeaterol_1	578.43351	578.4791	11.77
17	9-Hydroxy-12-oxo-10-octadecenoic acid	312.23006	312.232	9.38	40	Feruloy ampeaterol	578.43351	578.4856	12.21
18	12.13-Epoxy-9-octadecenoic acid	296.23514	296.2372	9.41	41	Docosanoic acid oxiranyl-methyl ester	396.36035	396.3616	12.33
19	Palmitic acid	256.24023	256.243	9.7	42	7-Stigmastenol-3-*O*-beta-D-glucoside	578.45464	578.4871	12.69
20	Isooleic acid	282.25588	282.2581	9.94	43	Yibeinoside C	739.45068	739.5078	13.08
21	Linoleyl acetate	308.27153	308.2731	10.11	45	TacroliMus monohydrate	803.48198	803.5584	13.99
22	1-Methyl-2-[(6*Z*.9*Z*)-6.9-pentadecadienyl]-4(1*H*)-quinolone	365.27186	365.2591	10.12					

#### 3.1.3. FT-IR analysis

**Fig. 3:**
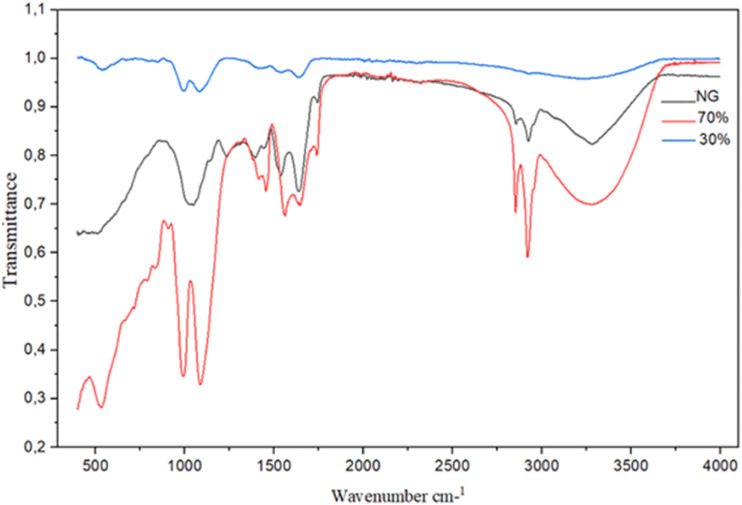
FTIR spectra of *N. sativa* seed powder and MgO-NPs.

FT-IR spectroscopy identified functional groups of diverse organic compounds based on the peak value in the region of 400–4000 cm^−1^. The presence of different-IR bands related to existences of various functional groups in *N. sativa* seed extract was notified (Fig. 3). For instance, peaks in 3377 and 2933 cm^−1^ related to O–H and aliphatic C–H stretching; the peaks at a range of 1417 and 1728 cm^−1^ correspond to C=C and C=O stretching, and peaks at 1255 and 1066 cm^−1^ could be ascribed to –C–O and –C–OC stretching. These peaks could be considered for the presence of various compounds such as phenolic, flavonoid, and carboxylic compounds.^28^ Two medium bands at 1075 cm^−1^ and 995 cm^−1^ also identified the presence of alkyl halid. Also, a band typical of Mg–O lattice vibration was noticed around 548 cm^−1^.

#### 3.1.4. Scanning electron microscope analysis (SEM)

**Fig. 4:**
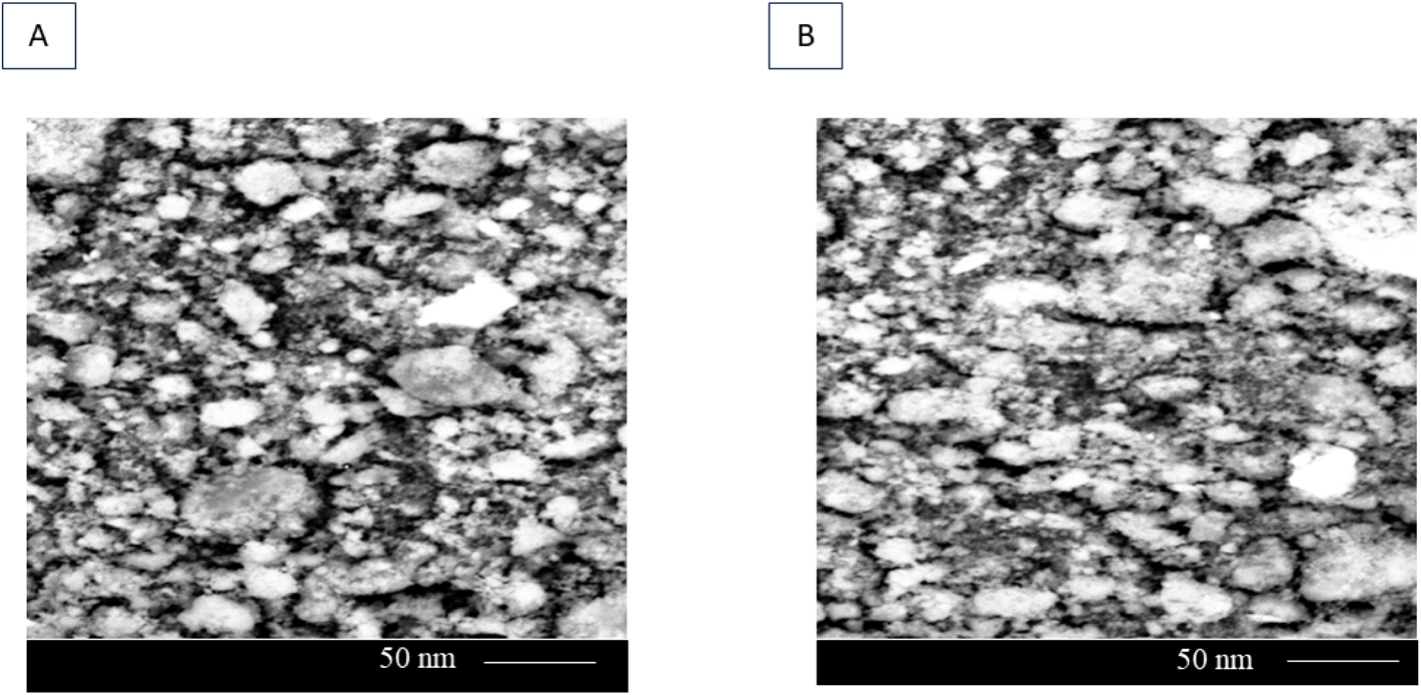
Visualization of green synthesized MgO-NPs by SEM. Visualization of green synthesized MgO-NPs by SEM (A and B).

The SEM micrographs revealed the successful formation of nanoparticles predominantly in the nanometer size range, with an average particle size of approximately 50–10 nm (Fig. 4A and B). SEM analysis demonstrated that the green-synthesized nanoparticles possess a relatively uniform morphology and nanometric dimensions. The presence of phytochemical constituents from *Nigella sativa* seed extract played a crucial role in regulating nucleation and growth, resulting in well-dispersed nanoparticles with limited aggregation. The surface morphology appeared smooth to slightly rough The particles exhibited a quasi-spherical morphology.

#### 3.1.5. Reducing power assay

**Fig. 5:**
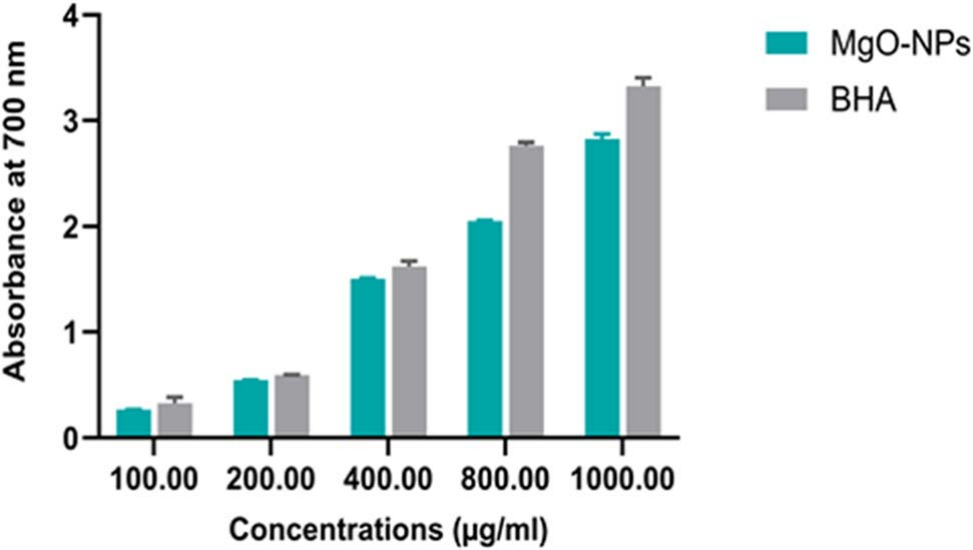
Reducing power capacities of MgO-NPs in comparison with butylated hydroxyanisole (BHA), used as standard.

In the reducing power assay, the antioxidant compounds lead to the reduction of ferric (Fe^3+^) to ferrous (Fe^2+^) forms due to their reductive capabilities. In this assay, the conversion of Fe^3+^/ferricyanide complex to the ferrous form was detected by the change of color from yellow to green or blue as shown in Fig. 5. MgO-NPs displayed a concentration-dependent manner, however, its activity is still lower than BHA.^29^

#### 3.1.6. ABTS˙^+^ scavenging assay

**Fig. 6:**
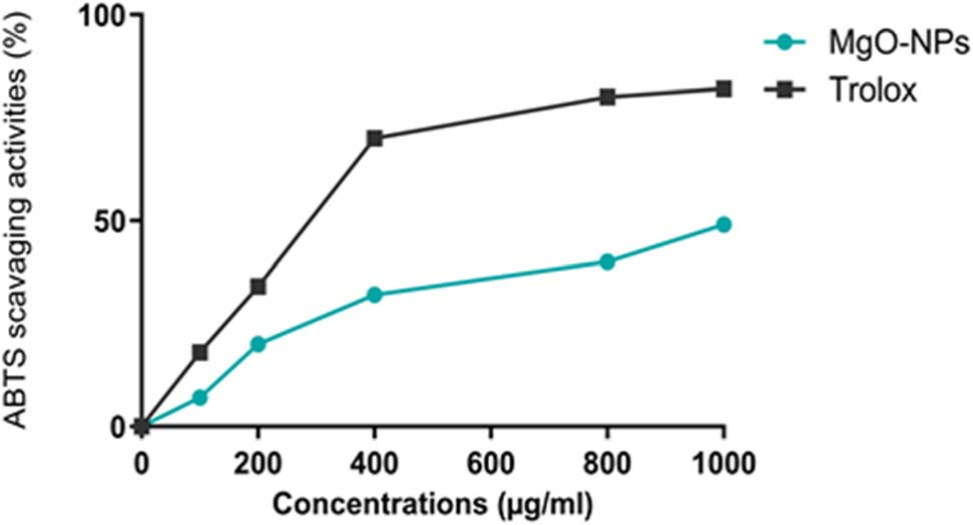
ABTS scavenging activities of Mg-NPs-NS in comparison with TROLOX, used as standard.

As illustrated in Fig. 6, the ABTS˙^+^ radical scavenging potential of MgO-NPs and standard trolox. The scavenging activities of the green MgO-NPs from *N. sativa* seed extract showed a direct relationship with the used concentration. At a concentration of 800 µg mL^−1^, MgO-NPs exhibited a scavenging activity reaching 50%, while Trolox displayed a scavenging activity of 80%. Trolox exhibited an IC_50_ value of 283.89 µg mL^−1^, whereas the IC_50_ values of MgO-NPs were determined as 800 µg mL^−1^.

### 3.2. *In vivo* study

#### 3.2.1. General characteristics of animals

Throughout the treatment period, no mortality was observed in any of the experimental groups, including the controls, STZ-treated rats, and STZ-treated rats with cutaneous excision wounds treated with MgO nanoparticles (MgO-NPs).

#### 3.2.2. Blood glucose level and body weight

During the study, a significant increase in blood glucose concentration in streptozotocin (STZ)-induced diabetic rats was observed compared to non-diabetic control rats throughout the experimental period. Notably, the application of green MgO-NPs resulted in a statistically significant decrease in blood glucose levels in the diabetic group measured at the study's endpoint. Conversely, STZ-induced diabetic rats exhibited a significant reduction in body weight compared to the control group during the experiment. However, treatment with MgO-NPs showed a trend toward normalization of body mass by day 17 post-wounding.

#### 3.2.3. Morphological and wound closure assessment

The complex interplay between diabetes and wound healing presents a significant clinical challenge, as chronic hyperglycemia fundamentally disrupts tissue repair. In the diabetic state, the initial inflammatory response often becomes prolonged and excessive. This sustained inflammation, coupled with an overproduction of reactive oxygen species (ROS), fosters a state of heightened oxidative stress, ultimately derailing the healing sequence and leading to delayed wound closure and other complications.^30^ The development of novel drug for wound care based on natural sources has garnered significant attention. Plants, rich in various bioactive phytochemicals which are widely used in traditional medicine to heal wounds. This study investigates the effectiveness of a 17 day topical application MgO-NPs, synthetized from *N. sativa* seed extract in promoting skin wound healing on STZ-induced type 1 diabetic rats.

**Fig. 7:**
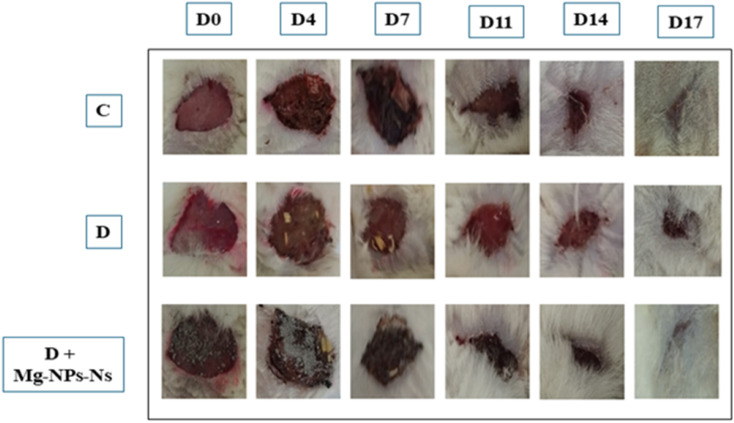
The wound-healing effect of MgO-NPs topical administration on excision wound model on different days (0, 4, 7, 11, 14, 17). C = controls; D = diabetes; D + MgO-NPs-NS = Diabetic rat + MgO-NPs.

The re-establishment of tissue integrity following injury is of utmost importance, with wound closure serving as a key indicator of successful healing. The selection of an excisional wound model for this 17 day study was predicated on its utility in demonstrating significant and measurable morphological changes throughout the healing process.^31^ Wound healing abilities were evaluated of green MgO-NPs on rats after circular excision in diabetic rats. The effectiveness of the green synthetized MgO-NPs on wound healing of diabetic rats was monitored for 17 days. As shown in Fig. 7, following the circular excision in skin, wound area measurements were performed before MgO-NPs treatment application on days 0, 4, 7, 11, 14, and 17. A rapid closure was observed in control group and STZ-MgO-NPs group. On the first day of the excision, all area wounds showed a bright red color. From the fourth day of treatment, the untreated wound showed an inflammatory response around over the damaged skin. The MgO-NPs-treated group (group 3) showed significantly faster recovery compared to both the negative control (group 1) and the STZ model group (group 2), as evidenced by a significant reduction in wound area measured on the 11th day. In addition, a brown color was showed in the control (group 1) and MgO-NPs-treated group due to the formation of the scab announcing the initiation of the healing process by the formation of blood clot (Fig. 7). The STZ-group showed persistent inflammation, indicated by the presence of a dark red coloration. On the 15th day, a complete closure of the wounds was observed in the STZ-group treated with MgO-NPs. Similarly, the untreated STZ group achieved full closure by this endpoint, despite showing a significantly slower healing rate throughout the study. These observations highlighted the healing role of MgO-NPs and its potential effect in acceleration wound healing mechanism. Our findings are in line with recent investigations on mice treated with hydrogel reinforced with green synthesized MgO nanoparticles as an effective wound dressing material.^32^

**Fig. 8:**
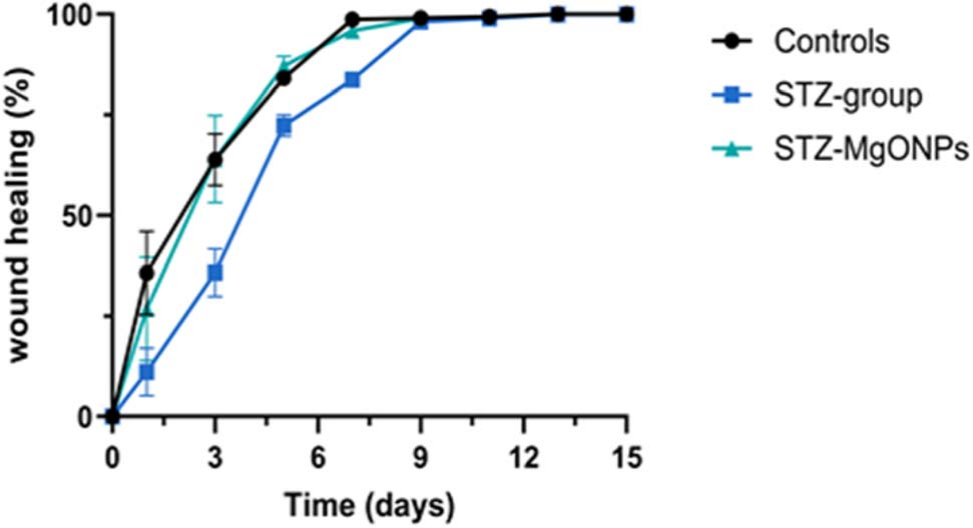
Evaluation curve of wound healing processes.

In the other hand, a quantitative evaluation of wound healing in all experimental groups is provided in Fig. 8. As the percentage of wound closure. The wound closure rates after 7 days were 98% in the control group and 95% in the STZ group treated with MgO-NPs, reflecting significant enhancement in epithelization and cell proliferation during healing. The wound closure percentage in the STZ group was 83% on the 7th day, indicating delayed healing of the excised wounds.

Our findings reveal that diabetic rats treated with MgO-NPs exhibited significantly enhanced wound closure ratios compared to both untreated diabetic and normal control groups after the 17 day treatment period. This result suggests that the green-synthesized MgO-NPs actively promote wound repair. As indicated by previous research, the therapeutic effect may be attributed to several mechanisms, including the modulation of inflammation and the direct enhancement of cellular repair processes in both normal and diabetic rats.^33^

#### 3.2.4. C-reactive protein (CRP) assessment

**Fig. 9:**
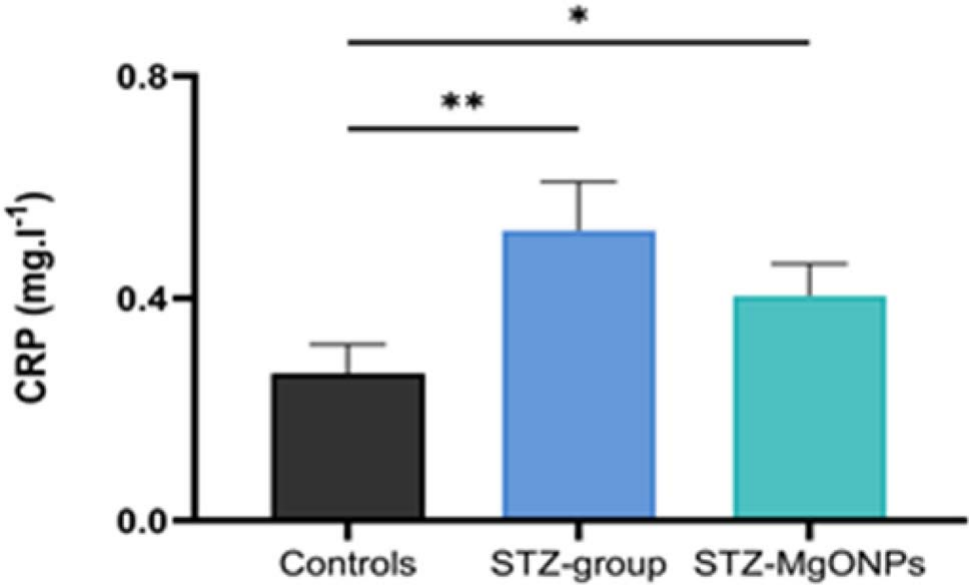
Effects of MgO-NPs application on C reactive protein (CRP) levels. Values are means ± SEM (*n* = 6). STZ group, STZ-Mg-NPs groups *vs.* control group:*p < 0.05; **p < 0.01.

CRP is a protein inflammatory marker, measured in the plasma of all treated rats. As illustrated in Fig. 9, plasma CRP levels were significantly elevated in STZ-treated rats (*F*_(2,9)_ = 14.57; *p* = 0.001) compared to the control group. Notably, the treatment with MgO-NPs in the STZ group was significantly reduced plasma CRP levels.

#### 3.2.5. Effects of MgO-NPs application on MDA and protein oxidation (AOPP) levels in wound healing

**Fig. 10:**
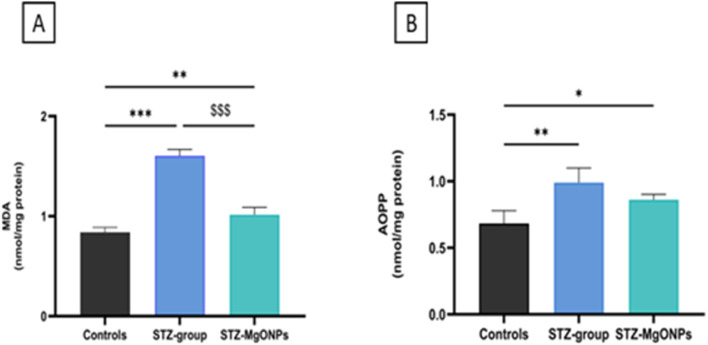
Effects of MgO-NPs application on MDA (A) and AOPP (B) levels in wound healing. Values are means ± SEM (*n* = 6). STZ-group, STZ-MgO-NPs groups *vs.* control group:*p < 0.05; **p < 0.01; ***p < 0.001. STZ-MgO-NPs groups *vs.* STZ-group: $$$ *p* < 0.001.

Previous investigations have consistently highlighted the prevalence of oxidative stress in diabetes,^34^ a condition that inflicts damage on vital cellular components like proteins, lipids, and DNA, ultimately leading to cell death and tissue dysfunction.^35^ The extent of this damage can be assessed by measuring the oxidation and carbonylation of specific amino acids providing a molecular signature of the detrimental impact of oxidative stress.^36^ The effect of magnesium nanoparticles on lipid peroxidation was assessed in the skin tissues of control and STZ-treated group by measuring MDA levels (Fig. 10A). A significant increase in MDA levels was observed in STZ-treated rats compared to the control group (*F*(_2,10_) = 160.8, *p* < 0.001). Diabetic rats with excised wounds treated with MgO-NPs showed a significant reduction in MDA levels (*p* < 0.006) compared to untreated controls. In addition, AOPP, recognized as end products of protein oxidation and served as crucial biomarkers to assess protein integrity and overall cellular health in wound healing. As shown in Fig. 10B, our results revealed a significant increase in the levels of AOPP in the healing tissue (*F*_(2,9)_ = 12.58, *p* = 0.002 of STZ-treated rats, when compared to controls. Interestingly, when MgO-NPs were applied locally in the excised skin of STZ-treated rats, there was a significant decrease (*p* = 0.04) in the levels of protein oxidation as compared to control group.

Our findings demonstrated that wounds in STZ-treated rats exhibited greater oxidative damage to lipids and proteins, as indicated by a significant rise in MDA and AOPP levels compared to the wound skin of the control group. However, MgO-NPs application to the excised skin significantly reduced both MDA and AOPP levels, suggesting a strong antioxidative effect. These results are consistent with previous reports showing that MgO-NPs synthesized *via* green methods—particularly using *N. sativa* seed extract enhance antioxidant defense by modulating ROS levels and restoring redox homeostasis.^37^ The observed reduction in oxidative stress biomarkers may also be attributed to the phytochemicals from *N. sativa*, which are known for their intrinsic free radical scavenging properties. Notably, palmitic acid contributes significantly to antioxidant effects by scavenging free radicals and its antibacterial effect.^38^ Kaempferol, a well-known secondary plant metabolite belonging to the flavonoids, exhibits an antidiabetic and antihypertensive compound as well as potent anti-inflammatory and antioxidant activities through inhibition of lipid peroxidation and enhancement of endogenous antioxidant enzymes.^39^ Additionally, thymoquinol glucoside, a bioactive quinone derivative, plays a crucial role in folk medicine to treat infections,^40^ reducing oxidative damage. Furthermore, our data align with findings from Khazaei *et al.*^41^ who demonstrated that MgO-NPs treatment leads to improved wound closure rates, collagen synthesis, and re-epithelialization in burn wounds models by attenuating oxidative and inflammatory damage. Collectively, these results reinforce the therapeutic potential of *N. sativa* seed extract mediated MgO-NPs in managing oxidative stress and promoting tissue regeneration in diabetic wound environments.

#### 3.2.6. Evaluation of wound healing antioxidant enzymatic activities and non-enzymatic oxidative stress markers

**Fig. 11:**
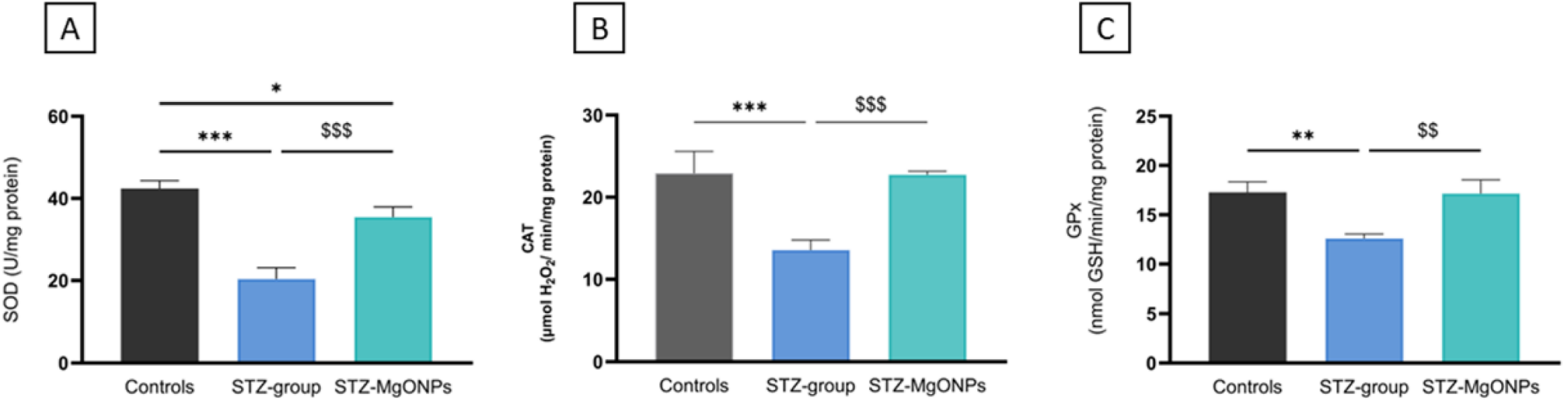
Effects of MgO-NPs application on SOD (A), CAT (B) and GPx (C) activities in wound healing. Values are means ± SEM (*n* = 6). STZ-group, STZ-Mg-NPs groups *vs.* control group:*p < 0.05; **p < 0.01; ***p < 0.001. STZ-MgO-NPs groups *vs.* STZ-group: $$ *p* < 0.01; $$$ *p* < 0.001.

Emerging recent research highlights the potential of MgO-NPs to modulate the activity of key antioxidant enzymes, which play a crucial role in the wound healing process, particularly under diabetic conditions. The use of *N. sativa* seed extract not only facilitates eco-friendly nanoparticle synthesis but also introduces bioactive compounds that possess antioxidant, anti-inflammatory, and antimicrobial properties, which contribute significantly to accelerating wound healing.^42^ The wound tissues superoxide dismutase (SOD), catalase (CAT) and glutathione peroxidase (GPx) activities were determined in all-treated rats and illustrated in Fig. 11A–C. On the 17th day after wounding, SOD, CAT, and GPx activities were significantly decreased in the STZ group (*F*(_2,8_) = 77.52, *p* < 0.001; *F*(_2,7_) = 35.13, *p* < 0.001; and *F*(_2,7_) = 18.38, *p* = 0.002, respectively) compared to the control group. Notably, following MgO-NPs application at the excised skin site, there was a significant restoration of antioxidant enzyme activities in the wounds compared to those in STZ-treated rats. In our study, the activities of antioxidant enzymes in the wound tissue of STZ-treated rats were significantly altered as compared to control rats throughout the healing process. Diabetic wounds showed consistently lower activity of antioxidant markers—SOD, GPx, and CAT—likely due to hyperglycemia-induced overproduction of ROS. Our findings agree with previous reports in diabetic models, where green-synthesized MgO nanoparticles prepared from medicinal plants improved antioxidant status and reduced oxidative damage, demonstrating the potential to ameliorate diabetic neuropathy in rats and suggesting a promising antidiabetic effect,^43^ enhance the healing of burn wounds^41^ and promote cell proliferation in an *in vitro* wound model.^44^ In line with these data, our study shows that *N. sativa* seed extract-mediated MgO-NPs markedly restored SOD, CAT, and GPx activities in the wound microenvironment, indicating efficient attenuation of oxidative stress. This pronounced normalization of antioxidant enzymes suggests that the rich phytochemical profile of *Nigella sativa* (including thymoquinone and polyphenols) may confer additional redox-modulating benefits compared with other plant-mediated MgO synthesis methods, thereby reinforcing the therapeutic potential of green-synthesized MgO nanoparticles particularly for diabetic wound management.

**Fig. 12:**
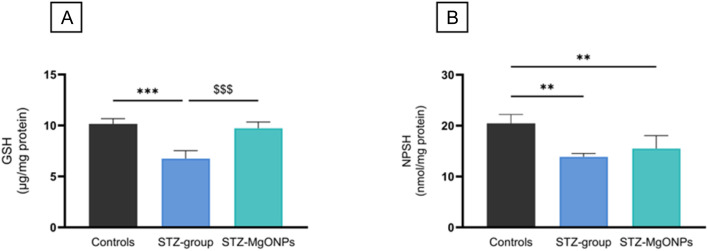
Effects of MgO-NPs application on GSH (A) and NPSH (B) levels in wound healing. Values are means ± SEM (*n* = 10). STZ-group and STZ-MgO-NPs group *vs.* Control group **p < 0.01; ***p < 0.001. STZ-MgO-NPs groups *vs.* STZ-group: $$$ *p* < 0.001.

By day 17 post-wounding, topical application of the green-synthesized MgO-NPs significantly increased the activity of those enzymes in diabetic wounds, suggesting a restorative effect on redox balance. Notably, this effect may also be attributed to the sustained release of Mg^2+^ ions from MgO-NPs synthesized using *N. sativa* seed extract, which have been shown to promote angiogenesis, cell proliferation, and tissue regeneration while mitigating oxidative stress and inflammation. This dual mechanism combining controlled magnesium ion release with the antioxidant properties of *N. sativa* seed extract phytochemicals positions MgO-NPs as promising therapeutic agents for improving impaired healing in diabetic wounds. These findings are consistent with previous reports showing that nanoparticle-based treatments, especially those derived from medicinal plants, can boost endogenous antioxidant responses and facilitate improved wound repair under oxidative stress conditions. This broad therapeutic potential is also demonstrated by alternative high-performance nanomaterials, such as black phosphorus (BP) platforms.^45^ While in the present study, MgO-NPs system promotes healing by scavenging ROS and enhancing natural antioxidant defenses, BP-based systems achieve therapeutic efficacy through generating localized heat and ROS. These effects synergistically disrupt bacterial membranes, induce oxidative damage, and inhibit bacterial metabolism. This effective sterilization reduces inflammation and promotes angiogenesis *via* VEGF and bFGF regulation, thereby accelerating diabetic wound healing.^45^ These distinct, yet equally powerful, approaches underscore the versatile role of nanomedicine in precisely modulating the oxidative stress environment for successful clinical outcomes in diabetes and other complicated diseases. Under diabetic conditions, oxidative stress is markedly prolonged, characterized by sustained expression of inflammatory cytokines, proteolytic enzymes, and excessive ROS that directly damage extracellular matrix proteins and impair cellular functions, ultimately hindering wound healing.^46,47^ Since antioxidant enzymes act synergistically with non-enzymatic antioxidants, reduced glutathione (GSH) and non-protein sulfhydryl groups (NPSH) levels were also evaluated in wound tissue of rats as shown in Fig. 12A and B. A significant decrease in GSH (*F*_(2,9)_ = 32.87, *p* < 0.001) and NPSH (*F*_(2,9)_ = 14.30, *p* = 0.002) levels in the skin tissue were observed in STZ-group when compared to those of control group. In our study, the skin of diabetic rats treated with MgO nanoparticles (MgO-NPs) exhibited significant increases in GSH and NPSH levels in wound tissues on day 17 after the wounding, suggesting a protective mechanism against oxidative damage during healing. One potential explanation for our findings is the ability of MgO-NPs to serve as a sustained-release source of Mg^2+^ ions within the wound microenvironment, maintaining optimal local concentrations without causing harmful rapid pH fluctuations or other adverse effects.^48^ This sustained release is crucial because Mg^2+^ acts as an essential cofactor for enzymes involved in antioxidant defense, such as those responsible for glutathione synthesis and regeneration, thereby supporting intracellular redox balance and neutralizing ROS.^49^ Furthermore, Mg^2+^ modulates key transcription factors, including Nrf2, which upregulate antioxidant enzyme expression, further strengthening cellular defenses and promoting tissue repair. Through these mechanisms, the controlled release of Mg^2+^ from MgO-NPs supports improved oxidative stress management and accelerates healing in diabetic wounds. This aligns with recent findings that MgO-NPs promote diabetic wound repair by suppressing inflammation, enhancing antioxidant defenses, and stimulating angiogenesis.^50^

#### 3.2.7. Histopathological examination

**Fig. 13:**
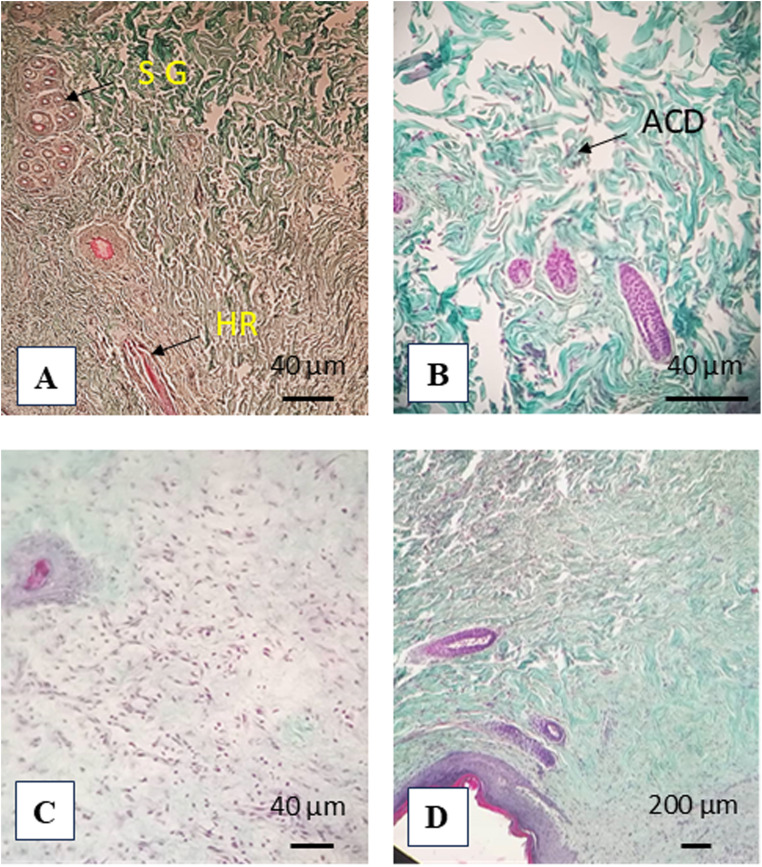
Control group: (A) arrow: sebaceous gland (SG), hair root (HR), normal collagen deposition. (B) STZ-group: abnormal collagen deposition (ACD). STZ-MgO-NPs groups (C and D): (C) significantly delayed rhythm of re-epithelialization and arrow: fibroblast formation was observed (D); full re-epithelization keratinization and hair follicles attachment for STZ-MgO-NPs groups. Modified trichrome staining.

The pattern of collagen deposition during the wound healing process was discovered by study utilizing modified trichrome staining. A dense and organized collagen matrix was seen in the control group, suggesting a normal skin tissue (Fig. 13). The Diabetic rat, displayed loosely distributed, uneven collagen fibers, which suggested that healing was either delayed or not completed. The presence of scarce spindle-shaped cells and disturbed collagen deposition suggested that fibroblast proliferation was delayed in diabetic tissue. For the STZ-MgO-NPs group showed a well-structured dermal architecture with dense, regularly spaced collagen fibers that were mostly stained with green, a sign of mature collagen deposition. With a decrease in inflammatory infiltrates and an increase in fibroblast activity, the epidermal layer seemed continuous and re-epithelialized, indicating efficient tissue remodeling and progressed wound healing.

## 4. Conclusion

In summary, physico-chemical characterizations combined with *in vitro* analyses and oxidative stress assessment provides a comprehensive framework for evaluating MgO-NPs particule. *In vivo*, histology shows fast and high-quality skin healing. Green synthesis of MgO-NPs incorporating *N. sativa* seed extract serve as an efficient delivery system for bioactive compounds, thereby promoting tissue regeneration in diabetic models.

## Ethics statement

All animals were treated in conformity with the General Guidelines on the Use of living Animals in Scientific Investigations (1986) and approved by the appropriate ethics committee for research involving humans and/or animals, under ethical approval no. 1205.

## Author contributions

Awatef Elwej: conception of the experiment; drug treatment, samples collection, analysis of biochemical parameters, histological pictures and interpretation, article preparation (text writing, figures and tables, statistical analysis); Abir Mhadhebi: experimental analysis, data analysis; Manel Mohammed Alzahrani: literature search, data acquisition; Hassane Oudadesse: manuscript editing and review; Hamadi Fetoui: review and editing manuscript, supervision; Naceur Mejri: physico-chemical analyses; Samira Jebahi: review and editing manuscript, supervision.

## Conflicts of interest

The authors have no competing interests.

## Data Availability

Data presented in this work will be provided upon reasonable request.
